# Longitudinal Relationship between Plasma Reactive Oxygen Metabolites and Periodontal Condition in the Maintenance Phase of Periodontal Treatment

**DOI:** 10.1155/2014/489292

**Published:** 2014-04-16

**Authors:** Tatsuya Machida, Takaaki Tomofuji, Daisuke Ekuni, Mayu Yamane, Toshiki Yoneda, Yuya Kawabata, Kota Kataoka, Naofumi Tamaki, Manabu Morita

**Affiliations:** ^1^Departments of Preventive Dentistry, Okayama University Graduate School of Medicine, Dentistry and Pharmaceutical Sciences, 2-5-1 Shikata-cho, Kita-ku, Okayama 700-8558, Japan; ^2^Department of Preventive Dentistry, Institute of Health Biosciences, The University of Tokushima Graduate School, 3-18 Kuramoto-cho, Tokushima 700-8503, Japan

## Abstract

*Aim*. The present cohort study describes the longitudinal relationship between plasma oxidative status and periodontitis progression during the maintenance phase of treatment. *Materials and Methods*. Forty-five patients (mean age 58.8 years) were monitored from 2008 to 2013. Periodontal conditions, including probing pocket depth (PPD) and clinical attachment level (CAL), were recorded. Measurements of plasma reactive oxygen metabolites (ROM) and biologic antioxidant potential (BAP) were performed to evaluate plasma oxidative status. The patients were assigned into 2 groups as low and high plasma ROM level using a cut-off value which was median of plasma ROM level at baseline. *Results*. In the subjects with low plasma ROM level at baseline, changes in mean CAL were positively correlated with changes in plasma ROM levels, bleeding on probing, and plaque control record, but not with PPD. In the subjects with high plasma ROM at baseline, changes in CAL were significantly associated with only PPD at baseline. On the other hands there were no significant associations between changes in CAL and those in plasma BAP levels. *Conclusions*. When plasma ROM level in periodontitis patients was low, increases in plasma ROM level were associated with those in CAL during the maintenance phase of treatment.

## 1. Introduction

Periodontitis is one of the most widespread chronic diseases and is characterized by gingival bleeding, periodontal pocket formation, destruction of connective tissue attachment, and alveolar bone resorption. The primary etiological agent for periodontitis is dental plaque bacteria [1, 2]. However, it is also recognized that the majority of periodontal tissue destruction is caused by abnormal host responses to these microorganisms and their products [3, 4].

Reactive oxygen species (ROS) is a collective term that includes superoxide, hydroxyl, and nitric oxide radical species, as well as nonradical derivatives of oxygen. Production of ROS is a normal part of cellular metabolism. However, overproduction of ROS disrupts the tissue oxidative/antioxidative balance, which induces oxidative injury by damaging DNA, lipids, and proteins [5]. Clinical studies have noted that periodontitis patients have elevated blood levels of total oxidative status [6, 7], lipid peroxidation [6], and protein oxidation [8] compared to periodontally healthy subjects. In addition, it has been shown that periodontal treatment decreases plasma oxidized low-density lipoprotein levels [9] and serum total oxidative status [10] in chronic periodontitis patients. Although it is still unclear whether oxidative status is the cause or result of periodontitis [10–12], it has been suggested that systemic increases in oxidative status can affect periodontitis progression.

Increases in oxidative status are involved in the progression of various diseases, including cardiovascular diseases [13], diabetes mellitus [14], liver diseases [15], and periodontitis [11, 16]. Furthermore, reviews have suggested that oxidative status acts as a potential common link that explains the relationship between periodontal and systemic conditions [17, 18]. Thus, measuring systemic oxidative status in periodontitis patients may be useful for evaluating the effects of systemic conditions on periodontal health.

The maintenance phase of periodontal treatment is important for maintaining a stable periodontal condition after initial preparation therapy, periodontal surgery, or therapy for recovering oral function. Unfortunately, periodontitis continues to progress in some patients, even during the maintenance phase of treatment. Since there is a positive correlation between changes in periodontal and systemic health [19–21], progressive periodontitis during the maintenance phase may also be associated with alterations in systemic health, including systemic increases in oxidative status. However, the longitudinal relationship between periodontal conditions and systemic oxidative status during the maintenance phase remains unclear.

Recently, a method for measuring reactive oxygen metabolites (ROM) has been found to be useful for the evaluation of oxidative status in the circulation [22–24]. This test measures the level of generic peroxide present in the plasma, which in turn reflects the level of free radicals from which they were formed. A close correlation between ROM levels and hydrogen peroxide concentrations in serum samples (*r* = 0.94, *P* < 0.001) supports the validity of measuring ROM to evaluate oxidative status [25]. Furthermore, as ROM measurement can be performed within 10 minutes, the feasibility of clinical monitoring of plasma ROM levels in periodontitis patients is high.

In the present study, we hypothesized that systemic increases in oxidative status might contribute to periodontitis progression during the maintenance phase of periodontal treatment. The purpose of this cohort study was therefore to investigate the longitudinal relationship between plasma ROM levels and periodontitis progression during the maintenance phase of therapy. To avoid the effects of the other systemic conditions on periodontitis progression, we excluded smokers and the subjects who had diabetes mellitus, liver diseases, cancer, dyslipidemia, lung diseases, and renal diseases. In addition, since aging [26] can affect oxidative status during a follow-up period, we examined the correlation between changes in periodontal condition and the continuous variables, including age. Furthermore, plasma levels of biologic antioxidant potential (BAP) were also measured to evaluate the corresponding antioxidative status [22, 27, 28]. This is a photometric test that measures the plasma biological antioxidant potential as the capacity of the plasma sample to reduce iron from ferric (Fe^3+^) to ferrous form (Fe^2+^).

## 2. Materials and Methods

### 2.1. Study Population

The patients participating in the present study were the same patient population (81 chronic periodontitis patients, 62 women and 19 men) characterized in our previous cross-sectional study [22] and were longitudinally monitored at the Department of Preventive Dentistry, Okayama University Hospital, from 2008 to 2013. All subjects received comprehensive dental care that included nonsurgical periodontal therapy consisting of oral examination, oral hygiene instructions, supra/subgingival debridement and scaling, and root-planning of all pockets (≥4 mm) every 3-4 months. At the onset of the study period (baseline), the patients had already entered the maintenance phase of therapy for more than 1 year (9.8 ± 7.6 years). Exclusion criteria were as follows: pregnancy, systemic diseases (such as diabetes mellitus, liver diseases, cancer, dyslipidemia, lung diseases, and renal diseases), previous or current smoking, users of antioxidant supplements, individuals with fewer than 15 teeth, use of anti-inflammatory medicine, and/or the presence of acute periodontal inflammation within the 3-month period prior to the start of the study period and final examinations. Due to several factors (26 moved away, 7 had medical problems, and 3 had data missing), 36 patients were lost prior to the follow-up examinations. As a result, data were analyzed for 45 subjects (41 women and 4 men, with a mean age of 58.8 years; response rate 55.6%). This study was approved by the Ethics Committee of Okayama University Hospital. After obtaining informed consent, the dentists completed a detailed medical questionnaire and subjects who fulfilled the study requirements were enrolled.

### 2.2. Oral Examination

Probing pocket depth (PPD) and clinical attachment level (CAL) were determined at six sites (mesiobuccal, mid-buccal, distobuccal, mesiolingual, mid-lingual, and distolingual) on all teeth using a color-coded probe (Hu-Friedy, Chicago, IL, USA). Sites that bled upon gentle probing (25 g probing force) were recorded, and the proportion of sites with bleeding on probing (BOP) was measured in each subject. The plaque control record (PCR) was measured using erythrosine staining and was recorded with respect to their relative location to the gingival margin at four sites (mesial, distal, buccal, and lingual) around each tooth [29]. All clinical procedures were performed by four trained and calibrated dentists (Takaaki Tomofuji, Yuya Kawabata, Daisuke Ekuni and Naofumi Tamaki). In order to assess intra- and interexaminer agreement, measurements of PPD and CAL were recorded and repeated within a 2-week interval for 8 volunteers randomly selected from the cohort of chronic periodontitis patients. Data were analyzed using the nonparametric *κ* test and intraclass correlation. The *κ* coefficients for intra- and interexaminer and intraclass correlation coefficients were >0.8.

### 2.3. Blood Sampling

Blood samples were obtained from the fingertip and immediately placed on ice. Following centrifugation at 3,000 ×g for 5 min, ROM levels were measured in the obtained plasma samples. Measurements of ROM levels were performed on the same day as blood sampling.

### 2.4. Measurement of Plasma ROM Levels

Plasma ROM levels were determined using a free radical evaluator (Diacron International, Grosseto, Italy), in accordance with previously reported procedures [22, 23].

The reaction utilized in this system is outlined below.

*Step* *1.* R–OOH (generic peroxide) + Fe^2+^ → R–O* (the alkoxyl radical of a generic peroxide) + Fe^3+^ + OH^−^ or R–OOH + Fe^3+^ → R–OO* (the peroxyl radical of a generic peroxide) + Fe^2+^ + H^+^ or R–O* + [Fe=O]^2+^ + H^+^.

*Step* *2.* R–O* or R–OO* + A–NH_2_ (N,N-diethyl-para-phenylendiamine [chromogenic substrate])→ R–O^−^ or R–OO^−^ + [A–NH_2_*]^+^ (colored).

[A–NH_2_*]^+^ is the colored radical cation of the chromogenic substrate that is spectrophotometrically detectable at 505 nm.

The results of measurements are expressed in arbitrary units (CARR U; derived from the name of the chemist [Carratelli] who invented the test), with 1 CARR U being equivalent to 0.08 mg/dL hydrogen peroxide.

The reliability of plasma ROM level determinations was confirmed by assessing plasma samples extracted three times every 4 hours from the fingertips (*n* = 18), with the intraclass correlation coefficient for an individual of 0.751. The same samples were also measured on three consecutive days for estimation of between-day reproducibility, and the coefficients of variation were <3%.

### 2.5. Measurement of Plasma BAP Levels

The plasma levels of BAP were also measured using a free radical electric evaluator (Diacron International, Grosseto, Italy), according to published analysis procedures [22, 27, 28]. A 10 *μ*L plasma sample is added to a solution of ferric chloride and thiocyanate derivate, and the intensity of any resulting decolourization is proportional to the ability of plasma to reduce ferric ions [30]. The results of the BAP test are expressed as *μ*mol/L of reduced iron. The intra-assay and the interassay coefficients of variation for this assay were <5%.

### 2.6. Statistical Analysis

We calculated mean PPD and mean CAL in each subject and used it for the following analysis. A paired* t*-test was used to compare the variables at baseline and 5-year follow-up. We divided the subjects with low or high oxidative status groups at baseline as follows: low (plasma ROM level ≤ 380 CARR U) or high (plasma ROM level > 380 CARR U). This cut-off value for the oxidative status was based on the median of the present results. A Fisher's exact test and an unpaired* t*-test were used to compare the variables at baseline of the subjects with low and high oxidative status.

Pearson's correlation coefficients were calculated to determine the correlation between changes in CAL and the other continuous variables (age, number of teeth present, PPD, CAL, BOP, PCR, ROM, and BAP) at baseline and the differences in the variables between baseline and the 5-year follow-up. Multiple linear regression analyses for all subjects and subjects with low or high oxidative status at baseline were also performed to evaluate the relationships between changes in CAL and the other variables. In order to control for confounding variables, the final model included variables that had a *P* < 0.2 in the bivariate analyses. We performed variance inflation factor analysis to detect and prevent multicollinearity between independent variables. Values of *P* < 0.05 were considered to represent statistically significant differences.

## 3. Results

Table 1 presents the characteristics of the study population. The proportion of female patients was higher than males. According to the paired* t*-test analyses, there were significant differences between baseline and 5-year follow-up in number of teeth present, CAL, teeth with CAL ≥7 mm, BOP, PCR, and BAP (*P* < 0.05). There were no significant differences in PPD and ROM (CARR U).

Comparisons of the subjects with low and high oxidative status at baseline are shown in Table 2. Unpaired* t*-test analysis indicated that the number of teeth present and CAL were significantly different in the two groups (*P* < 0.05).

Results of the Pearson's correlation coefficients between the changes in CAL and the other parameters are shown in Tables 3, 4, and 5. In the subjects with low oxidative status at baseline, the changes in CAL were significantly associated with changes in PPD (*P* < 0.05), BOP (*P* < 0.05), PCR (*P* < 0.05), and ROM (*P* < 0.05) (Table 4). In the all subjects and the subjects with high oxidative status at baseline, the changes in CAL were associated with PPD at baseline (*P* < 0.05) and the overall change in PPD (*P* < 0.05) (Tables 3 and 5).

The results of the multiple linear regression analyses are shown in Tables 6, 7, and 8. In the all subjects, changes in CAL were negatively correlated with PPD at baseline (*P* < 0.05) and positively correlated with PCR at baseline (*P* < 0.05) (Table 6). In the subjects with low oxidative status at baseline, changes in CAL were positively correlated with changes in BOP, PCR, and ROM (*P* < 0.05) (Table 7). In the subjects with high oxidative status at baseline, changes in CAL were negatively correlated with PPD at baseline (*P* < 0.05) (Table 8).

## 4. Discussion

To the best of our knowledge, this is the first epidemiological study to assess the longitudinal relationship between plasma ROM levels and periodontal progression during the maintenance phase of periodontal treatment. In this study, multiple linear regression analyses showed that changes in CAL are positively correlated with changes in plasma ROM levels after adjusting for changes in BOP and PCR in the subjects with low plasma ROM levels (≤380 CARR U) at baseline. ROM is an indicator of the oxidative status in the circulation [22–24]. When periodontitis patients exhibit low oxidative status, it is feasible that the elevation of oxidative status in the circulation could contribute to periodontitis progression during the maintenance phase.

At baseline, all subjects in this study had already received a supportive periodontal care program for more than 1 year. The low percentage of sites with BOP and dental plaque at baseline and final examinations reflected the characteristics of a well-maintained periodontal patient population. In this study, the results show that periodontitis progression was correlated with plasma ROM levels, even when low percentages of BOP and PCR were present. These suggest that periodontitis progression in the present population was associated with systemic risk factors for periodontitis. Animal studies have demonstrated that increases in serum ROM can lead to the progression of periodontitis [31, 32]. In addition, recent studies report that dietary habits influence serum ROM levels [33]. Although further studies are needed, therapeutic approaches exploiting alterations in dietary habits may offer clinical benefits to the prevention of periodontitis progression in patients during the maintenance phase of periodontal treatment.

Clinical studies have investigated the effects of antioxidant supplementation in periodontitis patients. For instance, it has been reported that supplementation of 8 mg/day lycopene produced a significant reduction in gingival bleeding when compared to controls [34]. A double-blind randomized controlled trial also showed that daily supplementation with a juice powder containing an antioxidant phytonutrient strengthened the effects of periodontal treatment on the reduction of PPD, relative to placebo [35]. These observations are consistent with the present results supporting the notion that systemic approaches to ROS reduction may be clinically effective in suppressing periodontitis progression.

In the subjects with high oxidative status (>380 CARR U) at baseline, there was no correlation between changes in CAL and changes in ROM. At baseline, periodontitis patients with high oxidative status had greater CAL than those with low oxidative status. The lack of correlation between changes in periodontal condition and ROM in periodontitis patients with high oxidative status might be due to the severity of periodontal disease already present at baseline.

Several studies have investigated the relationship between blood ROM levels and chronic diseases. For instance, clinical studies have demonstrated that high ROM levels could be a predictor of cardiovascular disease progression [36, 37]. It is also known that high ROM levels are an independent predictor of obesity (odds ratio: 2.5; *P* < 0.001) in women [38]. These observations show that increases in plasma ROM may contribute to the progression of chronic diseases. In this study, increases in plasma ROM levels were associated with changes in CAL in subjects with low plasma ROM levels at baseline. Although further studies are needed, it is possible that monitoring plasma ROM levels is a useful method for monitoring the effects of systemic conditions on periodontal health in periodontitis patients.

In our previous cross-sectional study [22], plasma ROM levels were positively associated with CAL in chronic periodontitis patients. We also found that nonsurgical periodontal treatment improved both periodontal inflammation and plasma ROM in chronic periodontitis patients [23]. Furthermore, the present findings indicated that the increased plasma ROM levels might contribute to changes in periodontal conditions. These results suggest that there is a close relationship between periodontal conditions and systemic oxidative status.

In this study, alterations in CAL were negatively correlated with PPD at baseline in subjects with high oxidative status. Recent research has suggested that baseline PPD is associated with subsequent alveolar bone density and height loss in postmenopausal osteopenic females undergoing periodontal maintenance [39]. However, it have been indicated that sites with deep PPD showed more attachment level gain after 3 months of scaling and root planning than those with shallow PPD [40]. In addition, it has been also reported that attachment level gain after 3 months of curettage of periodontal pockets was greater in deep PPD than in shallow PPD [41]. Since deep PPD may be affected by periodontal maintenance therapy to a greater extent than shallow PPD, the correlation between changes in CAL and PPD at baseline was negative in our study.

On the other hand, plasma BAP levels were not correlated with changes in CAL in this study. This indicates that the correlation between blood total antioxidant status and periodontitis progression is modest. Several methodologies are now available to evaluate blood antioxidant status, and a previous study suggested that the Fe^3+^ reducing antioxidant power assay used in our study has certain drawbacks, from the viewpoint of interference and reaction kinetics, in comparison with the oxygen radical absorption capacity (ORAC) method [42]. Therefore, the failure to observe a correlation between plasma antioxidative status and change in periodontal condition could be related to the choice of the BAP test. In a potential future study, additional assays (e.g., ORAC method) may be required to elucidate the correlation between systemic antioxidant status and change in periodontal condition.

An acknowledged weakness of this study is that all subjects were recruited at the Okayama University Hospital. This may limit the ability to generalize our findings to the broader population of patients in the maintenance phase of periodontal treatment. In addition, we did not use strict measures to control factors (C-reactive protein and dental biofilms) that might influence plasma ROM and periodontal conditions. In particular, it is reported that serum ROM levels are closely associated with serum high-sensitivity C-reactive protein in a Japanese population [43]. Measurement of high-sensitivity C-reactive protein would increase the validity of the presently established relationship between plasma ROM levels and periodontitis progression. In addition, the lack of annual follow-ups was also the limitation of the study because oxidative status may have not continued to simply increase or decrease.

## 5. Conclusion

In the subjects with low plasma ROM level (≤380 CARR U) at baseline, changes in mean CAL were positively correlated with those in plasma ROM levels during the maintenance phase of periodontal treatment. On the other hand, in the subjects with high plasma ROM level (>380 CARR U) at baseline, changes in mean CAL were not correlated with those in plasma ROM levels. Furthermore, there were no correlation between changes in mean CAL and those in plasma BAP levels.

## Figures and Tables

**Table 1:** Characteristics of the study population at baseline and 5-year follow-up.

Variables	Baseline (*N* = 45)	Follow-up (*N* = 45)	*P* value*
	*N* (%) or Mean ± SD	*N* (%) or Mean ± SD	
Gender			—
Male	4 (8.9)		
Female	41 (91.1)		
Age (years)	58.8 ± 10.1	64.6 ± 10.2	—
Teeth present (*n*)	25.0 ± 4.4	24.1 ± 4.9	<0.001
Periodontal parameters			
PPD (mm)	1.8 ± 0.3	1.9 ± 0.3	0.107
CAL (mm)	2.6 ± 1.1	2.8 ± 1.1	0.001
Teeth with CAL ≥7 mm (*n*)	1.2 ± 2.9	1.7 ± 3.6	<0.001
Sites with BOP (%)	4.5 ± 4.1	7.7 ± 7.6	0.012
PCR (%)	16.0 ± 13.4	21.3 ± 17.8	0.044
Plasma parameters			
ROM (CARR U)	390.6 ± 61.0	389.4 ± 63.8	0.913
BAP (*μ*mol/L)	2380.6 ± 244.8	1784.1 ± 501.5	<0.001

*Paired *t*-test.

*N*: number; SD: standard deviation; PPD: probing pocket depth; CAL: clinical attachment level; BOP: bleeding on probing; PCR: plaque control record; ROM: reactive oxygen metabolites; CARR U: Carratelli units; and BAP: biologic antioxidant potential.

**Table 2:** Comparisons of each parameter at baseline between the subjects with low oxidative status (≤380 CARR U) and high oxidative status (>380 CARR U).

Variables	Plasma ROM Level (CARR U)		*P* value
	Low oxidative status (*N* = 23) *N* (%) or Mean ± SD	High oxidative status (*N* = 22) *N* (%) or Mean ± SD	
Gender			0.608*
Male	3 (13.0)	1 (4.5)	
Female	20 (87.0)	21 (95.5)	
Age (years)	57.6 ± 9.4	60.1 ± 11.0	0.418^†^
Teeth present (*n*)	26.7 ± 3.0	23.2 ± 5.0	0.009^†^
PPD (mm)	1.8 ± 0.3	1.8 ± 0.4	0.959^†^
CAL (mm)	2.1 ± 0.4	3.0 ± 1.4	0.010^†^
Teeth with CAL ≥7 mm (*n*)	0.4 ± 0.6	2.1 ± 4.0	0.053^†^
BOP (%)	4.9 ± 4.3	4.2 ± 3.9	0.578^†^
PCR (%)	17.0 ± 13.9	15.1 ± 13.2	0.640^†^
BAP (*μ*mol/L)	2350.9 ± 230.0	2411.6 ± 261.0	0.412^†^

*Fisher's exact test. ^†^Unpaired *t*-test.

*N*: number; SD: standard deviation; PPD: probing pocket depth; CAL: clinical attachment level; BOP: bleeding on probing; PCR: plaque control record; ROM: reactive oxygen metabolites; CARR U: Carratelli units; and BAP: biologic antioxidant potential.

**Table 3:** Pearson's correlation coefficients between change of CAL and the independent variables in the all subjects (*N* = 45).

Independent variables	r* between ΔCAL and the independent variables	*P* value
Variables at baseline		
Age (years)	−0.058	0.707
Teeth present (*n*)	0.048	0.754
PPD (mm) at baseline	−0.427	0.003
CAL (mm) at baseline	−0.138	0.367
BOP (%) at baseline	−0.048	0.752
PCR (%) at baseline	0.240	0.112
ROM (CARR U) at baseline	0.134	0.381
BAP (*μ*mol/L) at baseline	0.004	0.982
Differences of variables		
ΔTeeth present (*n*)	−0.288	0.055
ΔPPD (mm)	0.583	<0.001
ΔBOP (%)	0.021	0.891
ΔPCR (%)	0.115	0.450
ΔROM (CARR U)	0.078	0.609
ΔBAP (*μ*mol/L)	−0.085	0.599

Δ represents differences (value at follow-up minus value at baseline).

*Pearson's correlation coefficients.

*N*: number; SD: standard deviation; PPD: probing pocket depth; CAL: clinical attachment level; BOP: bleeding on probing; PCR: plaque control record; ROM: reactive oxygen metabolites; CARR U: Carratelli units; and BAP: biologic antioxidant potential.

**Table 4:** Pearson's correlation coefficients between change of CAL and the independent variables in the subjects with low oxidative status (≤380 CARR U) (*N* = 23).

Independent variables	r* between ΔCAL and the independent variables	*P* value
Variables at baseline		
Age (years)	−0.082	0.711
Teeth present (*n*)	0.012	0.956
PPD (mm) at baseline	−0.268	0.217
CAL (mm) at baseline	−0.183	0.402
BOP (%) at baseline	−0.219	0.315
PCR (%) at baseline	0.305	0.157
ROM (CARR U) at baseline	0.062	0.778
BAP (*μ*mol/L) at baseline	0.038	0.863
Differences of variables		
ΔTeeth present (*n*)	−0.228	0.296
ΔPPD (mm)	0.660	0.001
ΔBOP (%)	0.563	0.005
ΔPCR (%)	0.487	0.018
ΔROM (CARR U)	0.443	0.034
ΔBAP (*μ*mol/L)	0.240	0.322

Δ represents differences (value at follow-up minus value at baseline).

*Pearson's correlation coefficients.

*N*: number; SD: standard deviation; PPD: probing pocket depth; CAL: clinical attachment level; BOP: bleeding on probing; PCR: plaque control record; ROM: reactive oxygen metabolites; CARR U: Carratelli units; and BAP: biologic antioxidant potential.

**Table 5:** Pearson's correlation coefficients between change of CAL and the independent variables in the subjects with high oxidative status (>380 CARR U) (*N* = 22).

Independent variables	r* between ΔCAL and the independent variables	*P* value
Variables at baseline		
Age (years)	−0.075	0.742
Teeth present (*n*)	0.152	0.500
PPD (mm) at baseline	−0.531	0.011
CAL (mm) at baseline	−0.228	0.308
BOP (%) at baseline	0.092	0.683
PCR (%) at baseline	0.233	0.298
ROM (CARR U) at baseline	0.032	0.887
BAP (*μ*mol/L) at baseline	−0.044	0.845
Differences of variables		
ΔTeeth present (*n*)	−0.352	0.108
ΔPPD (mm)	0.576	0.005
ΔBOP (%)	−0.235	0.293
ΔPCR (%)	−0.358	0.102
ΔROM (CARR U)	−0.075	0.741
ΔBAP (*μ*mol/L)	−0.256	0.249

Δ represents differences (value at follow-up minus value at baseline).

*Pearson's correlation coefficients.

*N*: number; SD: standard deviation; PPD: probing pocket depth; CAL: clinical attachment level; BOP: bleeding on probing; PCR: plaque control record; ROM: reactive oxygen metabolites; CARR U: Carratelli units; and BAP: biologic antioxidant potential.

**Table 6:** Multiple linear regression analysis with change of CAL as the dependent variable in the all subjects (*N* = 45).

Variables	*B* (95% CI)	*β*	*P* value
Intercept	1.068 (0.470, 1.666)	—	0.001
PPD (mm) at baseline	−0.534 (−0.853, −0.216)	−0.451	0.002
PCR (%) at baseline	0.008 (0.0003, 0.016)	0.262	0.042

We used backward elimination method with a *P* > 0.1 threshold for exclusion to construct the model. The candidate variables for the model were ΔTeeth present, PPD at baseline, and PCR at baseline. The *F*-statistic was 7.374 (*P* value was 0.002), the *R*-square was 0.260, and the adjusted *R*-square of the model was 0.225. The variance inflation factor was <5 and condition index was <15.

Δ represents differences (value at follow-up minus value at baseline).

*N*: number; CI: confidence interval; *B*: unstandardized regression coefficient; *β*: standardized coefficient; PPD: probing pocket depth; CAL: clinical attachment level; PCR: plaque control record.

**Table 7:** Multiple linear regression analysis with change of CAL as the dependent variable in the subjects with low oxidative status (≤380 CARR U) (*N* = 23).

Variables	*B* (95% CI)	*β*	*P* value
Intercept	0.044 (−0.060, 0.148)	—	0.391
ΔBOP (%)	0.013 (0.0003, 0.025)	0.349	0.045
ΔPCR (%)	0.006 (0.001, 0.010)	0.409	0.019
ΔROM (CARR U)	0.002 (0.0004, 0.003)	0.416	0.014

We used backward elimination method with a *P* > 0.1 threshold for exclusion to construct the Model. The candidate variables for the Model were ΔBOP, ΔPCR, and ΔROM. The *F*-statistic was 8.744 (*P*-value was 0.001), the *R*-square was 0.580, and the adjusted *R*-square of the model was 0.514. The variance inflation factor was <5 and condition index was <15.

Δ represents differences (value at follow-up minus value at baseline).

*N*: number, CI: confidence interval, *B*: unstandardized regression coefficient, *β*: standardized coefficient, CAL: clinical attachment level, BOP: bleeding on probing, PCR: plaque control record, CARR U: and Carratelli units.

**Table 8:** Multiple linear regression analysis with change of CAL as the dependent variable in the subjects with high oxidative status (>380 CARR U) (*N* = 22).

Variables	*B* (95% CI)	*β*	*P* value
Intercept	1.622 (0.650, 2.593)	—	0.002
PPD (mm) at baseline	−0.718 (−1.236, −0.200)	−0.539	0.009
ΔPCR (%)	−0.011 (−0.027, 0.006)	−0.251	0.193

We used backward elimination method with a *P* > 0.1 threshold for exclusion to construct the Model. The candidate variables for the model were ΔTeeth present, PPD at baseline, and ΔPCR. The *F*-statistic was 5.012 (*P* value was 0.018), the *R*-square was 0.345, and the adjusted *R*-square of the model was 0.276. The variance inflation factor was <5 and condition index was <15.

Δ represents differences (value at follow-up minus value at baseline).

*N*: number; CI: confidence interval; *B*: unstandardized regression coefficient; *β*: standardized coefficient; PPD: probing pocket depth; CAL: clinical attachment level; PCR: plaque control record; and CARR U: Carratelli units.
